# Noncleft Velopharyngeal Insufficiency: Etiology and Need For Surgical Treatment

**DOI:** 10.1155/2012/296073

**Published:** 2012-03-26

**Authors:** Steven Goudy, Christopher Ingraham, John Canady

**Affiliations:** ^1^Department of Otolaryngology, Vanderbilt University, Nashville, TN, USA; ^2^Department of Radiology, University of Washington, Seattle, WA, USA; ^3^Department of Otolaryngology Head and Neck Surgery, University of Iowa, Iowa City, IA, USA

## Abstract

*Objective*. Velopharyngeal insufficiency (VPI) occurs frequently in cleft palate patients. VPI also occurs in patients without cleft palate, but little is known about this patient population and this presents a diagnostic dilemma. Our goal is to review the etiology of noncleft VPI and the surgical treatment involved. *Design/Patients*. A retrospective review of VPI patients from 1990 to 2005. Demographic, genetic, speech, and surgical data were collected. We compared the need for surgery and outcomes data between noncleft and cleft VPI patients using a Student's *t*-test. *Results*. We identified 43 patients with noncleft VPI, of which 24 were females and 19 were males. The average age at presentation of noncleft VPI was 9.6 years (range 4.5–21). The average patient age at the time of study was 13.4 years. The etiology of VPI in these noncleft patients was neurologic dysfunction 44%, syndrome-associated 35%, postadenotonsillectomy 7%, and multiple causes 14%. The need for surgical intervention in the noncleft VPI group was 37% (15/43) compared to the cleft palate controls, which was 27% (12/43). There was not a statistical difference between these two groups (*P* > 0.5). *Conclusion*. Noncleft VPI often occurs in patients who have underlying neurologic disorders or have syndromes. The rate of speech surgery to address VPI is similar to that of cleft palate patients. We propose that newly diagnosed noncleft VPI patients should undergo a thorough neurologic and genetic evaluation prior to surgery.

## 1. Introduction

The velopharyngeal complex closes against the posterior pharyngeal wall, to separate the oral cavity from the nasal cavity. Inability of the velopharyngeal complex to close leads to nasal air emissions and hypernasal speech. The occurrence of velopharyngeal insufficiency (VPI) in the absence of cleft palate formation has been reported many times in the literature. VPI is a recognized complication of adenoidectomy [1–8]. Moreover, patients with velocardiofacial syndrome (VCFS) also have VPI without cleft palate [5, 9, 10].

 Individuals with suspected VPI are best evaluated by a team of professionals specializing in VPI and speech characteristics associated with cleft palate. Initially, each of these patients is evaluated by a speech pathologist to assess the patient's speech patterns, nasality, and articulation disorders [11]. From there, they will undergo evaluation of their velopharyngeal port closure using multiview fluoroscopy and/or video nasoendoscopy [12, 13].

 Once the diagnosis of VPI has been made a trial of speech therapy is often used to improve the intelligibility of speech. If speech remains unintelligible and/or VPI is a significant component of the speech pattern, surgical intervention is often needed. The type of surgery required is often based on the video nasoendoscopy [14]. The outcomes after speech surgery may vary based on the type of surgery performed and underlying medical diagnosis, especially in patients with VCFS [15, 16]. We hypothesize that patients with noncleft VPI require similar surgical management and have similar speech outcomes compared to cleft palate patients.

## 2. Materials and Methods

 After obtaining IRB approval, a retrospective review of patients charts diagnosed with VPI who did and did not have a cleft palate was performed. Patients with VPI treated at the University of Iowa Hospitals and Clinics from 1990 to 2005 were reviewed. Demographic, genetic, speech, and surgical data were collected. For each noncleft VPI patient we identified an age-and-sex matched control patient with a cleft palate. We compared the outcomes data using a Student's *t*-test. Briefly, each patient was evaluated according to the protocol by Dailey et al. for hyper and hyponasality (1 = normal, 2 = mild, 3 = mild-moderate, 4 = moderate, 5 = moderate-severe, 6 = severe) and underwent video nasal endoscopy [17]. Based on these findings, each patient was assessed to have a competent, marginally competent, or incompetent velopharyngeal closure. Patients judged to have marginally competent or incompetent velopharyngeal closure underwent surgical management of their VPI based on their port closure characteristics.

## 3. Results

 We identified 43 patients who had noncleft VPI. There were 24 females and 19 males found. The average age at the diagnosis of noncleft VPI was 9.6 years (range 4.5–21). The average age of the patient in the study was 13.4 years. The etiology of VPI in these noncleft patients was postadenotonsillectomy 7%, neurologic dysfunction 44%, syndrome-associated 35%, and unknown causes 14%.

The neurologic dysfunction group comprised the largest portion of the noncleft VPI group (44%). There were a wide spectrum of disorders included in this group detailed in Table 1, which includes posttraumatic brain injury, developmental delay, and cerebral palsy.

The occurrence of a syndrome in the noncleft patients with VPI was 35% and is shown in Table 2, which includes VCFS, Turner syndrome, and VATER syndrome. Of note, a new diagnosis of VCFS was made in 6 of our patients. The remaining group of patients did not have any definable risk factors for VPI.

The need for surgical intervention in the noncleft VPI group was 37% (15/43) compared to the cleft palate controls, which was 27% (12/43). In noncleft VPI patients, 10 pharyngeal flaps, 4 double-opposing Z plasties, and 1 sphincter pharyngoplasty were performed. In the cleft palate control group 11 pharyngeal flaps and 1 double-opposing Z plasty were performed. There was not a statistical difference in the number of VPI surgeries between these two groups (*P* > 0.5).

## 4. Discussion

 It is clear that noncleft VPI occurs in an assortment of anatomic, neurologic, postsurgical, and syndromic patients. A standard speech assessment is critical for making the diagnosis. The noncleft VPI patients were identified at a much later age, 9.6 years, than children with cleft palates. This is likely due to the speech screening that occurs in most cleft palate teams that identifies VPI earlier in children with cleft palate.

 Surgical intervention was required in 37% of noncleft VPI patients which is lower than what is reported in other studies [2, 3, 6]. The rate of VPI surgery did not statistically differ between noncleft and cleft palate patients. This suggests that noncleft VPI can be treated similar to cleft palate patients with initial speech therapy to improve articulation. However unresolved VPI required surgical intervention in more than a third of patients.

 It is important to note that an etiology of the VPI could be specifically identified in 86% of patients. Many of the patients had underlying neurological diagnoses or syndromes suggesting that altered neuromuscular control is associated with noncleft VPI. Newly diagnosed VCFS was identified in 16% of our patients, the importance of genetic evaluation has been highlighted by other authors [5].

## 5. Conclusion

 Noncleft VPI occurs in a diverse group of patients. The majority of these patients will not require surgical intervention. Each patient with noncleft VPI should be carefully screened for the etiology of their VPI because only 14% will have an unknown cause. The need for VPI surgery in noncleft patients is not different than cleft palate patients.

## Figures and Tables

**Table 1:** Neurologic causes of noncleft VPI.

Posttraumatic brain injury	*N* = 2
Developmental delay	*N* = 2
Cerebral palsy	*N* = 1
Myasthenia gravis	*N* = 1
Associated speech delay	*N* = 3
Undiagnosed neurologic condition	*N* = 10

**Table 2:** Syndromic causes of noncleft VPI.

Velocardiofacial syndrome	*N* = 7
Klippel-Feil syndrome	*N* = 1
Epidermal-Nevus syndrome	*N* = 1
Alagille syndrome	*N* = 1
Turner syndrome	*N* = 1
VATER syndrome	*N* = 1
Unrecognized syndrome	*N* = 1
